# Assessment of the Effect of Smartphone Usage on the Range of Motion and Fatigability of the Joints and Muscles of the Thumb Among Users: A Cross-Sectional Study in Central India

**DOI:** 10.7759/cureus.23199

**Published:** 2022-03-15

**Authors:** Ananyan Sampath, Avani Kulkarni, Revadi G, Manmohan Patel, Bertha A Rathinam

**Affiliations:** 1 Medicine, All India Institute of Medical Sciences, Bhopal, Bhopal, IND; 2 Anatomy, All India Institute of Medical Sciences, Bhopal, Bhopal, IND; 3 Community and Family Medicine, All India Institute of Medical Sciences, Bhopal, Bhopal, IND

**Keywords:** range of motion, fatigue, thumb, smartphone, goniometry

## Abstract

Introduction

The ability of adaptation is unique to humankind. Technology advances have introduced many appliances that increasingly are smaller in size and handheld. These devices on prolonged usage affect the thumb joint complex, and this study was therefore designed to assess any changes in the movement of the thumb joint complex and fatigability secondary to the increasing usage of smartphones in different orientations in the Central Indian population.

Materials and methods

An analytical cross-sectional study was performed to assess changes in the ranges of motion (ROM) of the thumb joint complex with a sample size of 137 selected nonrandomly and categorized on the basis of the orientation of smartphone usage by physical goniometer and a standardized questionnaire to assess fatigability.

Results

Most movements of the thumb joint complex corresponded to the existing standard values. The study found significant changes in the movement of passive flexion of the left metacarpophalangeal (MCP) joint and borderline significant modifications in the active extension of the left interphalangeal (IP) joint, passive extension of the left interphalangeal joint, and passive flexion of the left carpometacarpal (CMC) joint among the groups of participants. The Borg CR10 value of fatigue was “one,” indicative of no excessive fatigue after smartphone usage.

Conclusion

There are no significant changes in the ranges of motion of the joint complex of the thumb in mobile phone users over a period of time. The orientations and the increased duration of usage also did not cause any fatigue in the muscles of the thumb.

## Introduction

Evolution over the years has helped humankind as it is now to achieve greatness in the spheres of existence. It may have very well started with the presence of an opposable digit [1]. Among the primates, armed with the power of observance, consciousness, and insight, humankind came to establish dominance in the world.

Coming to the present era, humans have developed technology to simplify life, and one such major invention is the smartphone or mobile phone designed to be used mainly by our thumb, the opposable digit. Yet days have changed enough to involve our phones in everything. Individuals around the world have acquired this device at varying ages, some during their childhood and some only at late adulthood [2,3]. The 21st century has seen a constant increase in the number of smartphone users. By the end of 2017, there were 291.6 million smartphone users in India, with an expected growth rate of 16% year over year, which is the highest in the world [4]. According to press release number 16/2021 of the Telecom Regulatory Authority of India published on March 17, 2021, there are a total of 1,163.41 million wireless subscribers in India (until January 2021), with a monthly growth rate of 0.84% [5].

During text messaging, the thumb covers 79% of the maximum range of motion (ROM) in the adduction/abduction plane and 55% of its maximum range of motion in the flexion/extension plane, positioning the thumb in extreme postures, thereby placing an unfavorable static load on the intrinsic and extrinsic musculature of the thumb [6-10].

The movements of the thumb joint complex have undergone changes with the excessive use of smartphones. Recent researches emphasize the negative effects of mobile phone usage. Its effect on vision [11], the posture of the neck [12-15], dependence, addiction, and psychological distress on withdrawal [11,16] have been studied extensively.

To date, several studies have been undertaken to assess the effect of smartphone usage on fatigability [14] and strain on the muscles of the back [12-15], neck, and upper limb [12,13]. Moreover, a study has been conducted by Trudeau et al. to check for the enhancement of one movement of the thumb joint complex over the others [6].

Studies identifying the most ideal typing keyboard size [17,18] and ideal angles of flexion have also been done [7], and studies exploring the effect of typing on the movements of the thumb joint complex and identifying the ideal grip and orientation have been undertaken in the recent past [17-19]. Studies trying to identify the subjective symptoms experienced by the participants pertaining to smartphone usage have also been undertaken [14].

No study, to the best of our knowledge, has ever been devised to find out the most suitable position and orientation for smartphone usage comparing fatigability and range of motion via goniometric analysis in the Central Indian population. Therefore, the present study was planned to assess the fatigability and change in the range of motions in the joints of the thumb (pollex) among the various groups of smartphone users based on the orientation of the screen and duration of usage.

## Materials and methods

Study design and sample size

An analytical cross-sectional study was designed to assess the changes in the range of motion on the thumb joint complex (carpometacarpal (CMC), metacarpophalangeal (MCP), and interphalangeal (IP) joints) among 137 regular smartphone users of both sexes from a city in Central India. The participants were selected nonrandomly from Bhopal, Madhya Pradesh, after informed written consent.

Sample size

Assuming the large effect size of 0.40, probability of alpha error of 0.05, and 80% power of the study, with a partial eta^2^ of 0.5, grading the exposure in four groups and number of covariant 1, the sample size was calculated to be 111 with aid of G (power version 3.1.9.2). The present study collected data from 137 participants.

Categorization

Based on the orientation of the phone screen, the participants were divided into four groups (Table 1).

**Table 1:** Depicting the various orientations and laterality of smartphone usage

Group	Orientation	Laterality of the hand
A	Portrait	Unilateral thumb – dominant hand
B	Portrait	Unilateral index – dominant hand
C	Portrait	Bilateral thumbs
D	Landscape	Bilateral thumbs

Portrait refers to the long axis of the smartphone or the longer side stands parallel to the long axis of the hand, whereas, in a longitudinal perspective, the long axes are perpendicular to each other.

The data collected from the participants included an array of both independent and dependent variables. The independent variables collected are shown in Table 2.

**Table 2:** Independent variables under consideration

Independent variables collected	Particulars
Participant particulars	Name
	Age (in years)
	Sex
General examination	Height (in cm)
	Weight (in kg)
	Blood pressure (in mm of mercury)
	Pulse (in beats per minute)
	Temperature (in degree Celsius)
Basic clinical tests of the manus	Tinel’s test
	Two-point discrimination
	Phalen’s test
	Grind’s test
	Finkelstein’s test
	Froment’s test
	Paper holding test
Responses from a closed-ended, previously validated questionnaire	Borg CR10 for fatigue perception

Basic clinical examinations of the hand, which included Tinel’s test, two-point discrimination, Phalen’s test, Grind’s test, Finkelstein’s test, Froment’s test, and paper holding test, were all done according to Orthobullet’s guidelines for examination of the hand [20]. Those with any history of injury to the manus and specific occupation that involves the use of smartphones were excluded from the study.

The fatigue perception was tested using a closed-ended questionnaire developed and validated by Dr. Gunnar Borg and Dr. Elisabet Borg and was used with permission from the BorgPerception AB company for the purposes of this academic research [21].

The dependent variables, which are the manual goniometric measurements across the thumb joint complex (carpometacarpal flexion, extension, opposition, and abduction, metacarpophalangeal flexion, and interphalangeal flexion and extension) in both active and passive modes bilaterally were recorded.

Statistical analysis

Data entry was done in Microsoft Excel 2010 (Microsoft Corp., Redmond, WA, USA), and data were coded and analyzed using the base R software version 4.1.1 and R packages available in open domains. Proportions and median (IQR) were calculated for the nonparametrically distributed data. Chi-square test/Fisher’s exact test was used to compare proportions among groups and test associations appropriately. The Kruskal-Wallis sum test was performed to test the association between more than two categorical groups and the independent numerical predictors. P-value < 0.05 was considered to be significant.

Ethical consideration

The research proposal was approved by the Institutional Human Ethics Committee (IHEC) after a recommendation from the Department of Anatomy, All India Institute of Medical Sciences (AIIMS), Bhopal (permission number IHEC-LOP/2021 / IM0382).

## Results

Our study included 137 participants, of which the majority 85 (62%) belonged to group C, followed by 31 (23%) in group A, 15 (11%) in group D, and six (4.4%) in group B.

Table 3 provides the distribution of age, gender, characteristics of smartphone users (years of smartphone usage and hours of daily use), and size of the screen against the different orientation groups of smartphone usages.

**Table 3:** Participant characteristics stratified group-wise *Results as median **Results as number of participants

Characteristics		Overall (N = 137)	Group A (N = 31)	Group B (N = 6)	Group C (N = 85)	Group D (N = 15)
Age^*^		20	19	42	20	20
Sex**	Female	71	19	2	48	2
	Male	66	12	4	37	13
Years of use^*^		3	3	10.50	3	4
Hours of use^*^		5	5	5	5	10
Size of screen**	≦5.5 inches	3	1	0	2	0
	5.5–6 inches	23	6	2	15	0
	6–6.5 inches	54	13	3	32	6
	≩6.5 inches	57	11	1	36	9

The overall median (IQR) age group of the participants was 20 (19,21) years. Nearly half of the participants in the study were females 71 (52%). The overall median (IQR) years of smartphone usage was three (2,5) years in almost all the groups except in group B and group D, where there was comparatively longer usage. Similarly, the median (IQR) of smartphone usage in hours was comparable in all the groups except group D with 10 (6,10.5) hours of phone use. A total of 57 (42%) participants used smartphones with a screen size of more than six and a half (6.5) inches.

The range of movements across the thumb joint complex was tabulated as angles for all joint movements except opposition, which was measured as the distance in centimeters. The difference in median (IQR) for different movements and different groups for significance was studied using the Kruskal-Wallis sum test (Table 2). The difference in the median (IQR) values of left passive flexion of the metacarpophalangeal (MCP) joint among different groups (P = 0.035) was found to be significant, whereas borderline significance was observed in left active flexion MCP (P = 0.051). Similarly, the difference in the median (IQR) values of left active extension IP (P = 0.074), left passive extension IP (P = 0.060), and left passive flexion CMC (P = 0.072) among the four groups showed borderline significance (Table 4).

**Table 4:** Distribution of the range of motion (ROM) of different joints across various groups as median (IQR) All values as measured in degrees. CMC: carpometacarpal joint, MCP: metacarpophalangeal joint, IP: interphalangeal joint

Characteristics	Overall (N = 137)	Group A (N = 31)	Group B (N = 6)	Group C (N = 85)	Group D (N = 15)	P-value
Right active flexion CMC	17 (14,20)	17 (15.5,20.5)	14.5 (14.0,18.8)	17 (14,20)	16 (14,21.5)	0.6
Right passive flexion CMC	19 (16,22)	19 (16.5,22)	18 (16,21.5)	19 (16,22)	20 (15.5,24.5)	0.9
Left active flexion CMC	18 (15,20)	17 (15,19.5)	14.5 (13.2,18.8)	18 (15,20)	18 (15,23)	0.7
Left passive flexion CMC	19 (17,22)	19 (17,20.5)	18 (17.2,18.8)	19 (16,22)	22 (20,25.5)	0.072
Right active extension CMC	45 (35,50)	45 (37,48)	39 (33,52)	43 (35,50)	42 (36,54)	>0.9
Right passive extension CMC	49 (42,57)	50 (44,55)	56 (39,70)	47 (41,57)	45 (42,56)	0.8
Left active extension CMC	48 (40,55)	50 (43,55)	48 (35,52)	46 (39,55)	48 (38,52)	0.7
Left passive extension CMC	57 (48,63)	56 (51,62)	62 (61,66)	57 (48,63)	57 (48,63)	0.4
Right active abduction CMC	70 (59,75)	70 (65,75)	50 (40,62)	70 (55,80)	71 (64,88)	0.10
Right passive abduction CMC	80 (70,92)	80 (74,91)	78 (54,86)	76 (70,92)	90 (78,96)	0.2
Left active abduction CMC	72 (58,85)	75 (60,85)	76 (69,80)	73 (58,85)	68 (44,85)	0.6
Left passive abduction CMC	85 (68,96)	84 (70,93)	99 (81,108)	81 (67,95)	85 (69,98)	0.4
Right active flexion MCP	55 (47,65)	55 (46,60)	58 (55,62)	55 (46,65)	65 (51,74)	0.12
Right passive flexion MCP	65 (57,75)	62 (55,72)	64 (62,65)	65 (57,75)	69 (59,79)	0.5
Left active flexion MCP	54 (45,63)	50 (40,58)	62 (62,62)	55 (46,63)	59 (45,68)	0.051
Left passive flexion MCP	62 (54,72)	55 (48,67)	68 (64,76)	64 (56,72)	68 (53,75)	0.035
Right active flexion IP	85 (76,90)	85 (80,90)	86 (80,88)	84 (74,90)	87 (75,92)	0.8
Right passive flexion IP	92 (84,100)	92 (84,98)	90 (85,92)	91 (83,103)	94 (90,98)	>0.9
Left active flexion IP	84 (75,88)	85 (75,90)	80 (75,86)	82 (74,87)	85 (77,91)	0.5
Left passive flexion IP	90 (81,96)	90 (85,98)	91 (83,93)	90 (81,96)	94 (84,96)	>0.9
Right active extension IP	16 (8,28)	17 (10,28)	20 (19,22)	15 (7,27)	27 (12,36)	0.4
Right passive extension IP	17 (8,28)	17 (10,28)	22 (20,22	15 (7,27)	27 (12,41)	0.3
Left active extension IP	13 (7,21)	13 (8,20)	20 (15,22)	13 (5,20)	21 (14,42)	0.074
Left passive extension IP	14 (7,21)	13 (8,20)	20 (15,24)	13 (5,20)	21 (14,56)	0.060

Spearman’s correlation was performed between parameters such as years of smartphone use and range of movements, of which the left active flexion of MCP was found to be negatively correlated (r = -0.21, P = 0.01). No significant correlation was observed between any of those.

Fatigue perception as measured by the overall Borg CR10 score was “one” (1 (0,3)), which was the same for all groups except group B where the score was “two” (2 (0.2,3)).

## Discussion

In our study, we observed that the ranges of motion of different joints of the thumb have shown mild variations from the preset standards [22-28]. This is probably due to the varied duration and orientation of usage of the smartphone and other extraneous variables and lifestyle. The variations are not entirely significant in all the joints of the thumb joint complex except in the first left interphalangeal joint.

According to the study conducted by Norkin and White [9] and Barakat et al. in 2013 [10], the following are the preset means of the ranges of motion for the thumb joint complex (Table 5).

**Table 5:** Comparison of the ranges of motion of the thumb joint complex with similar studies *Our study values are depicted as active/passive values for each joint motion. AAOS: American Academy of Orthopaedic Surgeons, AMA: American Medical Association

		AAOS [22,23]	AMA [24]	Jenkins [25]	De Smet [26]	Yoshida [27]	Skvarilová and Plevková [28] (active/passive)	Our study (right)*	Our study (left)*
CMC	Abduction	70°					70°	68.3°/81°	71°/82°
	Flexion	15°					15°	17°/20°	18°/20°
	Extension	20,80°	35°	59 (11)°	54 (13.7)°		8.1°/15°	43°/49°	47°/56°
MCP	Flexion	50°	60°			77°	57°/67°	56°/65°	54°/62°
	Extension	0°	40°	67 (11)°	79.8 (10.2)°	35°	13.7°/22.6°		
IP	Flexion	80°	80°			81°	79.1°/ 85.8°	82°/91°	80°/88°
	Extension	20°	30°			33°	23.2°/34.7°	17°/19°	19°/20°

The American Academy of Orthopaedic Surgeons in 1965 [22] and 1994 [23] arrived at the normative values of ranges of motion as shown above. The data observed in our study are similar in abduction and flexion of the carpometacarpal joint, flexion of the metacarpophalangeal joint, and flexion and extension of the interphalangeal joints bilaterally but showed differences in extension of the carpometacarpal joint. Very similar trends are observed in both active and passive movements bilaterally in the study by Skvarilová and Plevková [28].

The American Medical Association [24] in their study of thumb joint movements also arrived at similar degrees for the range of motion in movements of the metacarpophalangeal joint and flexion at the interphalangeal joint but showed a slight difference of ±10° in interphalangeal joint extension and carpometacarpal extension.

In the studies conducted by Jenkins et al. [25] and De Smet et al. [26], the values of the extension of carpometacarpal joints differ by a similar margin with our study.

Yoshida et al. [27] conducted a similar study in 2003; the values of metacarpophalangeal flexion and interphalangeal extension differ by a few degrees, whereas the interphalangeal flexion values are concordant with our study.

The lack of standardization of manual goniometric values does not lead to conclusive inferences of a definite change, yet studies conducted by Ellis and Bruton [29] and Bear-Lehman and Abreu [30] do suggest that a margin of error of 5° for manual goniometric analysis of the thumb, which accounts for most similarities. The differences can also be explained by the fact that the study population of the previous studies have included different populations and performed in a different time period; thus, a direct comparison between the studies may not be explicit. Studies have also inferred differences in the range of motion (ROM) between different sexes and different age groups only among manual goniometric analysis, yet as most of the abovementioned studies do include similar age ranges and almost equal distribution of sexes in our study, these factors are less likely to influence any difference that exists [26,29].

With the results as stated above, we can see that there has been little or no change in the present scheme of the ranges of motion as offered by the thumb joint complex among the users of smartphones who participated in the study.

## Conclusions

The cross-sectional study compared the range of motion and fatigability across different groups, with group C having the most number of individuals. The mean range of movements we have observed for the active flexion of the metacarpophalangeal joint of the left hand was 54°(P = 0.051) and 17° for the left interphalangeal joint (P = 0.071).

The Borg CR10 value of fatigue showed an overall score of “one,” indicative of no excessive fatigue after smartphone usage in the sampled population.

There are no significant changes in the range of motion of the joint complex of the thumb in mobile phone users over a period of time. The orientations and the increased duration of usage also did not cause any fatigue in the muscles of the thumb. Electromyographic studies and evaluating the fatigue after mobile usage for a longer duration might provide future application in terms of best orientation and maximum duration of usage, beyond which there might be pathological changes in the joint complex of the thumb.
